# Rapid, Sensitive and Simultaneous Detection of Two Wheat RNA Viruses Using Reverse Transcription Recombinase Polymerase Amplification (RT-RPA)

**DOI:** 10.3390/life12121952

**Published:** 2022-11-22

**Authors:** Zhiqing Chen, Tianye Zhang, Jiajia Lei, Ziqiong Wang, Peng Liu, Kaili Zhong, Jianping Chen, Jiaqian Liu

**Affiliations:** 1 State Key Laboratory for Quality and Safety of Agro-Products, Institute of Plant Virology, Ningbo University, Ningbo 315211, China; 2 College of Plant Protection, Nanjing Agricultural University, Nanjing 210095, China

**Keywords:** wheat yellow mosaic virus, Chinese wheat mosaic virus, viral detection, RT-RPA

## Abstract

In China, wheat yellow mosaic disease is mostly caused by wheat yellow mosaic virus (WYMV) and Chinese wheat mosaic virus (CWMV). If wheat is co-infected with these two viruses, it can cause severe yellow mosaic symptoms and yield losses. Early detection of viruses is crucial for preventing disease in the field. In this study, we optimized a sensitive, specific reverse transcription recombinase polymerase amplification (RT-RPA) detection method for two viruses, WYMV and CWMV. Two sets of primers were designed based on the capsid protein (CP)-encoding genes of the two viruses, and the reaction conditions were determined. The RT-RPA method, which amplified the target amplicon by a handheld reaction mixture for 20 min, was more sensitive than PCR-CP in the detection of WYMV. Finally, the RT-RPA method was performed on 110 randomly selected field samples, demonstrating its applicability to samples from different regions and specificity for co-infected samples. This study not only describes an improved method for detecting WYMV and CWMV using RT-RPA but also demonstrates the potential of this method, which could be applied under field conditions.

## 1. Introduction

In China, wheat yellow mosaic disease, which is mostly caused by wheat yellow mosaic virus (WYMV) and Chinese wheat mosaic virus (CWMV), can cause significant yield losses. Hence, it is one of the most important wheat diseases affecting production [1,2]. Since the 1970s, this disease has been recorded in Anhui, Shandong, Jiangsu, Shanxi, Zhejiang, Henan, Hubei and Sichuan Provinces in China [3]. Infected wheat plants develop yellowing or chlorotic streaks on their leaves, show stunted growth before heading [4] and yield reductions up to 10–30%, or even up to 70% in severely diseased fields [5].

In the field, if wheat is co-infected with WYMV and CWMV, it causes severe disease symptoms and yield losses [3]. Plants that are already infected with CWMV are more susceptible to infection by WYMV, which may aggravate the disease [6]. Both viruses are transmitted by the soil-borne plasmodiophorid parasite *Polymyxa graminis* [7]. CWMV, a member of the genus *Furovirus*, *Virgaviridae family* [8], consists of two positive-sense single-stranded RNA1 and RNA2. CWMV RNA1 (~7.2 Kb) encodes three proteins including the replication-associated protein, RNA-dependent RNA polymerase (RdRp) and a movement protein. CWMV RNA2 (~3.6 Kb) encodes four proteins involving a major capsid protein (CP), two minor CP-related proteins (N-CP and CP-RT) and a cysteine-rich protein (CRP) [6,8]. WYMV belongs to the genus *Bymovirus*, *Potyviridae* family [9]. It also comprises two positive-sense single-stranded RNAs. WYMV RNA1 (~7.6 Kb) encodes eight proteins named the third protein (P3), 7-kDa peptide (7K), cylindrical inclusion protein (CI), 14-kDa peptide (14K), viral protein genome-linked (VPg), nuclear inclusion-a protease (NIa-Pro), nuclear inclusion “b” protein (NIb) and coat protein (CP) [9]. WYMV RNA2 (~3.5 Kb) encodes two mature proteins, namely P1 and P2. CP is a relatively conserved protein among strains of the same virus [4,10]. Furthermore, as the CP gene is often used for viral detection, it could be considered when developing the RT-RPA method for detecting viruses [8,11,12,13].

Early detection of pathogens is crucial when aiming to prevent pathogens from spreading and to ensure food security [14]. Many methods of detecting CWMV and WYMV have been reported to date, such as electron microscopy (EM) [2,15], enzyme-linked immunosorbent assays (ELISAs) [6,16], reverse transcription polymerase chain reaction (RT-PCR), quantitative RT-PCR (qRT-PCR) [17,18], and reverse transcription loop-mediated isothermal amplification (RT-LAMP) [19]. EM has played a crucial role in providing morphological information about these plant virions [20,21]. Although ELISA is sensitive, it requires high-quality antisera, which is costly and time-consuming [22]. RT-PCR and qRT-PCR are commonly used for detecting plant RNA viruses [2,23,24,25,26]; however, they are both labor-intensive and time-consuming and require specific operating technology [27]. RT-LAMP is an isothermal amplification, but its application is limited by the fact that it requires four or more primers, and the primers are complicated to design [28]. With the progress of isothermal amplification, recombinase polymerase amplification (RPA) technology was developed by Piepenburg and colleagues in 2006 [29]. RPA has the advantages of high sensitivity, rapid amplification and time-saving and convenient operation [28]. In recent years, RT-RPA technology has been applied to detect many plant viruses, such as barley yellow dwarf virus [12], cucurbit chlorotic yellows virus [30], apple chlorotic leaf spot virus [31], potato virus Y [32] and chilliveinal mottle virus [33]. Remarkably, to date, no study has reported the development of an RPA method to detect CWMV and WYMV.

Imitating the T4 bacteriophage nucleic acid replication mechanism, RPA of target fragments in vitro is achieved by a recombinase loading factor (T4 uvsY) and three enzymes: a recombinase (T4 uvsX), a single-stranded DNA binding protein (SSB) (GP 32) and a strand-displacing polymerase (Bsu polymerase) [28,29]. During the reaction, in the presence of ATP, recombinase–primer complexes scan the template DNA and locate in a homologous sequence. After strand exchange, SSBs bind single-stranded DNA and stabilize the structure. Then, strand-displacing polymerases lengthen the primer [28]. The T4 uvsY works on the recombinase–primer complexes assembly [29]. The nucleic acid amplification reaction can be completed in 20 min at a constant low temperature of 25–43 °C [32,34,35]. In this way, the exponential amplification of nucleic acids can be accomplished. As for RNA viruses, RPA is combined with a reverse transcriptase appropriate for detecting RNA virus genome. 

In this study, we investigated the appropriate experimental conditions for the application of RPA technology to the detection of WYMV and CWMV. Furthermore, the method was tested and validated using naturally infected samples from diseased fields. We expect that the optimization of this method will provide technical support for the early diagnosis, monitoring and identification of wheat virus diseases.

## 2. Materials and Methods

### 2.1. Virus and Plant Material

All the wheat leaf samples were collected from plants with symptoms of wheat yellow mosaic disease in the major wheat-producing areas of China, including Anhui, Shandong, Henan, Shanxi and Jiangsu provinces. Two batches of wheat samples were collected in 2021 and 2022. The plant samples were stored at –80 °C. 

### 2.2. Total RNA Extraction and cDNA Synthesis

Total RNA was extracted from the fresh leaves using the HiPure Plant RNA Mini Kit (Magen, R4151-03) following the manufacturer’s operating instructions, and then resuspended in 60 μL of RNase-free water. Next, cDNA was synthesized using the First Strand cDNA Synthesis Kit (TOYOBO, FSK-101) according to the manufacturer’s instructions, and RNA quantitation was performed to obtain a concentration of 100 ng/μL. Then, the product was used as the template for PCR and RPA.

### 2.3. PCR Assays

For WYMV and CWMV detection, PCR assay was carried out to identify samples with a single infection, co-infected and healthy samples. We used primer pairs WYMV-CP-F/WYMV-CP-R and CWMV-CP-F/CWMV-CP-R to detect the CP of CWMV and WYMV, and the lengths of amplified fragments were 530 bp and 283 bp, respectively (Table S2) [2]. In accordance with the instructions of the Green Taq Mix (Vazyme, P131-AA), a PCR system was configured and the following program was set up: 95 °C for 5 min; 35 cycles of 95 °C for 30 s, annealing for 30 s at 56 °C for CWMV and at 58 °C for WYMV, and then 72 °C for 40 s, with a final extension at 72 °C for 10 min. For barley yellow mosaic virus (BSMV) and wheat steak mosaic virus (WSMV) detection, we used 2 × Phanta Max Master Mix (Vazyme, P515-AA), primers (Table S2) and cDNAs to amplify a partial sequence of the CP-encoding gene of BSMV and polyprotein-encoding gene of WSMV. The PCR products were inserted into pCB301 vector (GenBank accession number JN029690) plasmid for specificity detection. 

### 2.4. Primer Design 

The CP-encoding gene sequences of CWMV and WYMV were used as the query sequences in a nucleotide blast search (https://blast.ncbi.nlm.nih.gov/Blast.cgi, accessed on 30 June 2021), and homologous sequences were downloaded from NCBI (https://www.ncbi.nlm.nih.gov/, accessed on 30 June 2021). In total, five nucleotide sequences were found for CWMV CP and 100 nucleotide sequences for WYMV CP, and then sequence identity analysis was performed for the development of primers in conserved regions (Table S1). All nucleotide sequences were used to design common primers on the NCBI primer blast website (https://www.ncbi.nlm.nih.gov/tools/primer-blast, accessed on 30 June 2021). Primers with conserved sequences were selected according to the multi-sequence alignment result of DNAMAN. According to the manufacturer’s instructions (https://www.twistdx.co.uk/support/rpa-assay-design/, accessed on 30 June 2021), for each virus detection, four primer pairs (30–35 nt in principle) [14] were initially selected by different amplifications (approximately 150 bp, 250 bp, 350 bp and 450 bp), and the best pairs were used in the following experiments.

### 2.5. Recombinant Plasmids and Viruses 

First-strand cDNA was synthesized from the total RNA using ReverTra Ace-α-^®^kit (TOYOBO, FSK-101) and then used to amplify cDNA sequences of the CP-encoding gene of BSMV and the polyprotein-encoding gene of WSMV by performing PCR with 2 × Phanta Max Master Mix (Vazyme, P515-AA). The resultant products comprising the intact sequence of CP of BSMV and polyprotein of WSMV were digested with BamHI/SacI and ligated into the BamHI/SacI-digested plasmid pCB301 vector, resulting in recombinant plasmids comprising CP sequence of BSMV and polyprotein sequence of WSMV, respectively.

### 2.6. RPA Reaction

The RPA reaction was carried out using the TwistAmp Basic Kit (TwistDx, TABAS03KIT). The 25 μL total reaction volume contained 1 μL each of forward primer and reverse primer (10 μM), 14 μL of rehydration buffer and 5.75 μL RNA-free water, 2 μL of template and 1.25 μL of magnesium acetate (280 mM). The reaction system was well mixed after 4 min at a constant temperature and then incubated for another 16 min. The reaction was terminated at 95 °C for 5 min [35]. The product was purified using a GeneJET Gel Extraction Kit (Thermo Scientific, K0692) and analyzed by 1.5% agarose gel electrophoresis.

### 2.7. RT-RPA Reaction Optimization

Firstly, to select the best RPA primers, three samples with a single virus infection, one healthy sample and one plasmid (pCB-35S-R2 of CWMV [15] or pCB-35S-R1 of WYMV [2], which were stored in our laboratory and used as a positive control) were used in the primer selection experiment. Next, a positive sample was randomly selected to optimize the reaction temperature and time. The temperature gradient of 20 °C, 30 °C, 35 °C, 40 °C and 45 °C and the incubation time gradient of 5 min, 10 min, 20 min and 30 min were set. For testing the adaptability of RPA under a crop field environment, handheld and water bath incubation conditions were investigated. The handheld (a human hand, temperature nears to 37 °C) incubation was performed by holding the reaction tube in hand for a period of time. Water bath incubation was carried out in a portable thermos cup filled with 40 °C warm water for a period of time. 

### 2.8. Sensitivity Assay

cDNA synthesized from 100 ng/μL RNA was used as the initial template concentration, and then serially diluted 10-fold (10 to 10^−5^ ng/μL) to assess the sensitivity of three different methods, including PCR using CP primers (PCR-CP, with a product of 283 bp for WYMV and 530 bp for CWMV), PCR using RPA primers (PCR-RPA, amplified 346 bp product for WYMV and 437 bp for CWMV) or a RT-RPA using RPA primers (RT-RPA). The sensitivity levels of these assays were compared through analysis in 1.5% agarose gel electrophoresis.

### 2.9. Specificity Assay

According to the optimal reaction conditions obtained in single virus detection, we attempted to establish a specific method for the simultaneous detection of WYMV and CWMV by adding two viral RPA primer pairs to the reaction system. Three samples with a single infection of either WYMV or CWMV were used. As none of the wheat samples had a single infection of CWMV, *Nicotiana benthaminana* plants positive for CWMV were used for the RPA specificity assay. Three co-infected samples, a healthy sample and a plasmid mixture of the two viruses were used to determine the feasibility of the RPA method for detecting co-infections. In order to verify the specificity of RT-RPA with two sets of primers, we also tested the plasmids of BSMV and WSMV.

### 2.10. Applicability Assay

To test the applicability of the RPA method, the natural samples collected from Henan, Shanxi, Anhui, Shandong and Jiangsu provinces were used. RNA extraction, cDNA synthesis and product analysis were performed as previously described. The viruses were detected in these samples through a handheld RT-RPA in 20 min. We measured the agreement rate between RT-RPA and standard PCR in terms of virus detection using a kappa value [36], as well as the sensitivity and specificity using MedCalc application software (MedCalc software Ltd., Ostend, Belgium).

## 3. Results

### 3.1. Samples Screened by PCR-CP

**Figure 1:**
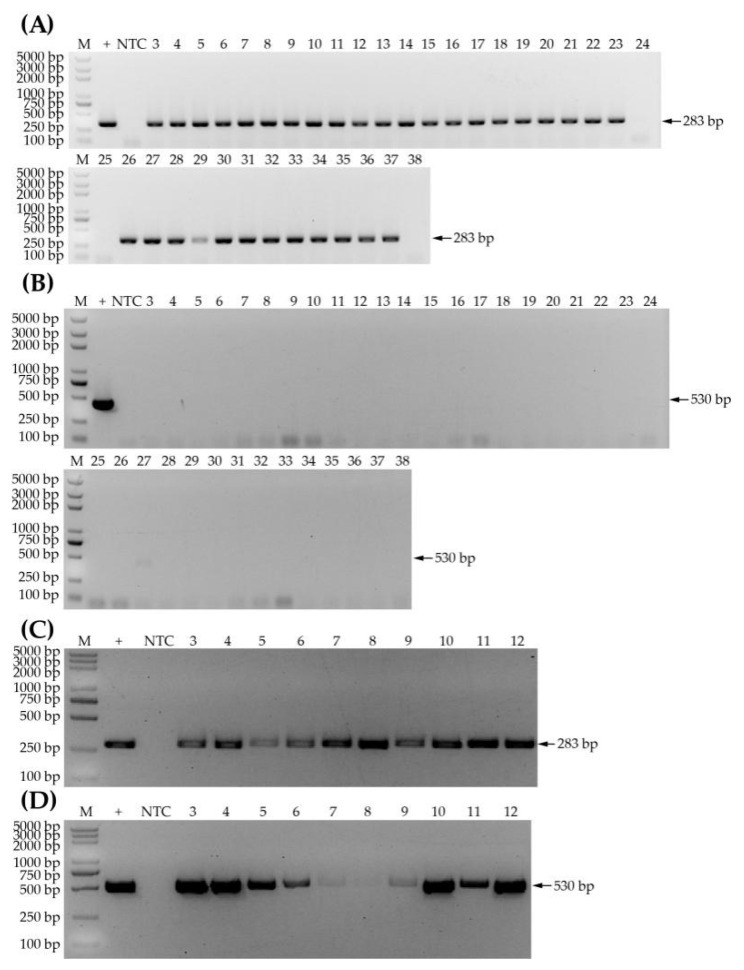
Samples screened by PCR using CP-specific primers. (**A**) Detection of a single infection of WYMV. Lane M, D2000 Plus DNA ladder; lane +, positive control; lane NTC, non-template control; lanes 3–11 (samples HN-1 to HN-8) and lanes 26–38 (samples HN-9 to HN-22) are from Henan province; lanes 12–25 (samples SD-1 to SD-14) are from Shandong province. (**B**) Detection of a single infection of CWMV. Samples are arranged in the same order as those in (**A**). **(C)** Detection of WYMV for co-infected plants. Lanes 3–6 (samples SD-15 to SD-18) are from Shandong province; lanes 7–12 (samples AH-1 to AH-6) are from Anhui province. (**D**) Detection of CWMV for co-infected plants. Samples are the same as those shown in (**C**). The CWMV plasmid pCB-35S-R2 and the WYMV plasmid pCB-35S-R1 were used as templates of positive controls for the detection of the corresponding virus. The D2000 Plus DNA ladder was used as a marker throughout the study.

The first naturally infected samples were screened for WYMV and CWMV infection by RT-PCR using primers of CP conserved sequences. The result showed that the specific bands of WYMV but no bands of CWMV were amplified in 33 samples (i.e., HN-1 to HN-21 and SD-1 to SD-12), and neither WYMV nor CWMV could be detected in three samples (i.e., samples SD-13, SD-14 and HN-22) (Figure 1A,B). In addition, fragments of the two viruses were detected in 10 samples (i.e., SD-15 to SD-18 and AH-1 to AH-6) (Figure 1C,D). Therefore, this screen revealed that 33 samples were infected with WYMV; three samples, not infected with either CWMV or WYMV, could be healthy; and 10 samples were co-infected with CWMV and WYMV, which could be used in further experiments. Hence, we randomly picked three positive samples and one healthy sample (i.e., HN-6, SD-3, AH-2 and SD-13, respectively, for WYMV detection, and SD-15, SD-17, AH-5 and HN-22, respectively, for CWMV detection), which were used for selecting RPA primers. 

### 3.2. Selection of RPA Primers

**Figure 2:**
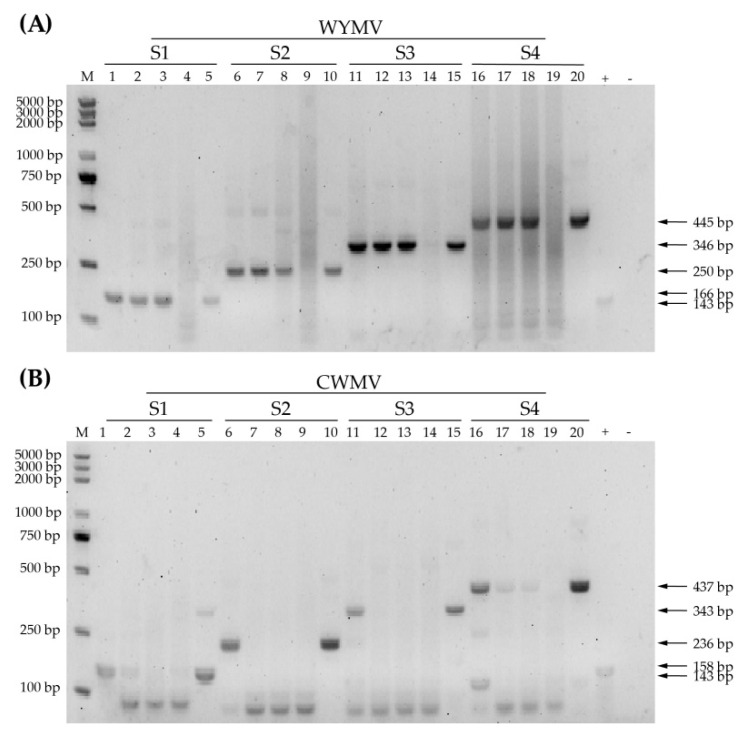
Selection of RPA primers. (**A**) RPA products of four sets of WYMV RPA primers. Five samples were used for each primer set: three infected samples and one healthy sample screened by PCR-CP as well as one plasmid, displayed in that order. + and − are positive and negative controls provided by the Twist Amp^TM^ Basic Kit (TwistDx, TABAS03KIT). (**B**) RPA products of four sets of CWMV RPA primers. The sample order is the same as that in (**A**).

The first step in optimizing RPA detection is selecting the best primer set. To this end, RPA reactions using different primers (Table 1) and the same templates were performed at 39 °C for 20 min according to the manufacturer’s instructions. WYMV-S3 produced the clearest and sharpest bands and a 346 bp amplicon was generated (Figure 2A). WYMV-S3 was therefore deemed the most suitable primer for WYMV detection. Among the CWMV primers tested, amplification with CWMV-S4 showed a distinct target band in all three positive samples and plasmid template (Figure 2B). The sizes of target amplicons were different between WYMV-S3 and CWMV-S4, which makes it easier to differentiate WYMV and CWMV in multiplex RPA. To summarize, WYMV-S3 and CWMV-S4 were chosen for use in the following investigation. 

### 3.3. Optimization of Incubation Temperature and Time

**Figure 3:**
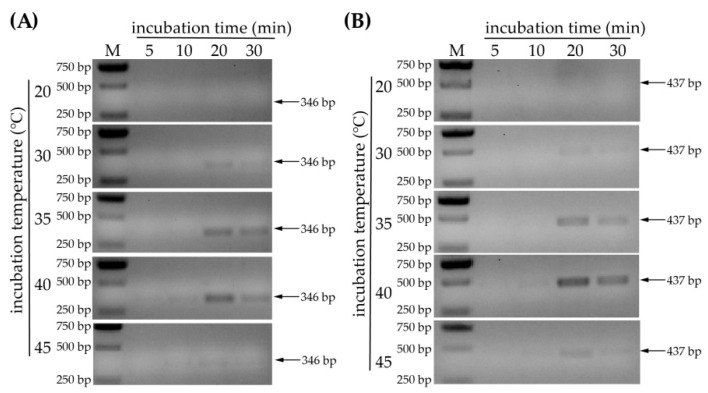
Optimization of reaction conditions by determining the most suitable incubation temperature and time. (**A**) Optimization of WYMV detection using a temperature gradient of 20 °C, 30 °C, 35 °C, 40 °C and 45 °C and an incubation time gradient of 5 min, 10 min, 20 min and 30 min. (**B**) Optimization of CWMV detection (temperature and time gradients are the same as those in (**A**)).

In addition to using suitable primers, the optimum temperature and incubation time also need to be adjusted to optimize the reaction. For this purpose, time gradients of 5 min, 10 min, 20 min and 30 min and temperature gradients of 20 °C, 30 °C, 35 °C, 40 °C and 45 °C were set as reaction conditions. The optimized conditions for WYMV detection showed that no target amplicon was detected at 20 °C. The band of expected size (346 bp) was observed following incubation at 30 °C or 35 °C for 20 min (Figure 3A). Furthermore, it is noticeable that amplicons were detected only after 10 min of incubation at 40 °C or 45 °C, and the band obtained at 45 °C was obviously weaker than that obtained at 40 °C (Figure 3A). In addition, the bands did not become more prominent with longer incubation time (Figure 3A). The same experiment was performed for the detection of CWMV, and the results were similar to those obtained for WYMV (Figure 3B). Therefore, we concluded that incubation temperatures of 35 °C or 40 °C and an incubation time of 20 min were the most suitable reaction conditions for amplicon detection. These reaction conditions were finally used for subsequent experimental development of the RPA system.

### 3.4. Validation of Incubation Conditions under Simulated Field Conditions

**Figure 4:**
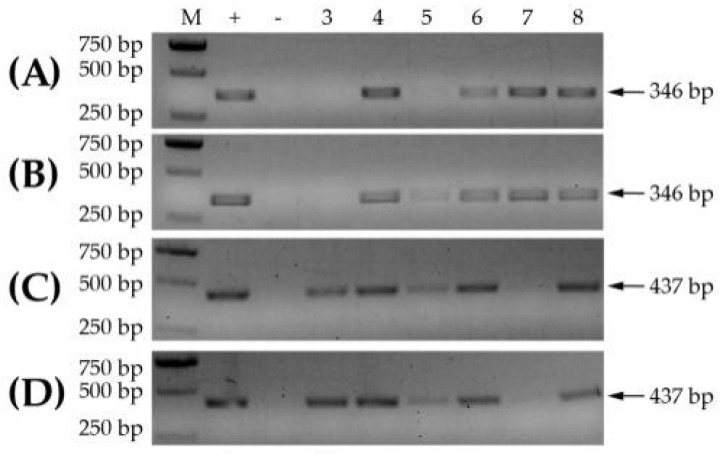
Validation of incubation conditions for handheld or 40 °C water bath condition. (**A**) RPA detection of WYMV at handheld condition. Lane M, D2000 Plus ladder; +, positive control; −, negative control; lanes 3 to 8 are samples SD-1, SD-3, HN-13, SD-17, SD-18 and AH-1, respectively. (**B**) RPA detection of WYMV at 40 °C water bath condition. Samples used are the same as those in (**A**). (**C**) RPA detection of CWMV at handheld condition. Lanes 3 to 8 are samples SD-15, SD-17, SD-18, AH-4, AH-5 and AH-6, respectively. (**D**) RPA detection of CWMV at 40 °C water bath condition. Samples used are the same as those in (**C**).

In order to make the detection conditions more widely applicable in the field, according to the more optimal reaction conditions of 35 °C or 40 °C for 20 min, the reaction temperatures of RT-RPA were set at about 37 °C for the handheld and at a 40 °C water bath under simulated conditions. The RT-RPA detection results of either WYMV or CWMV in the different samples were similar between the handheld and the 40 °C water bath (Figure 4). Thus, RPA detection of viruses would be performed in the field using the more convenient handheld.

### 3.5. Sensitivity Determination by Comparing PCR-CP, PCR-RPA and RT-RPA

**Figure 5:**
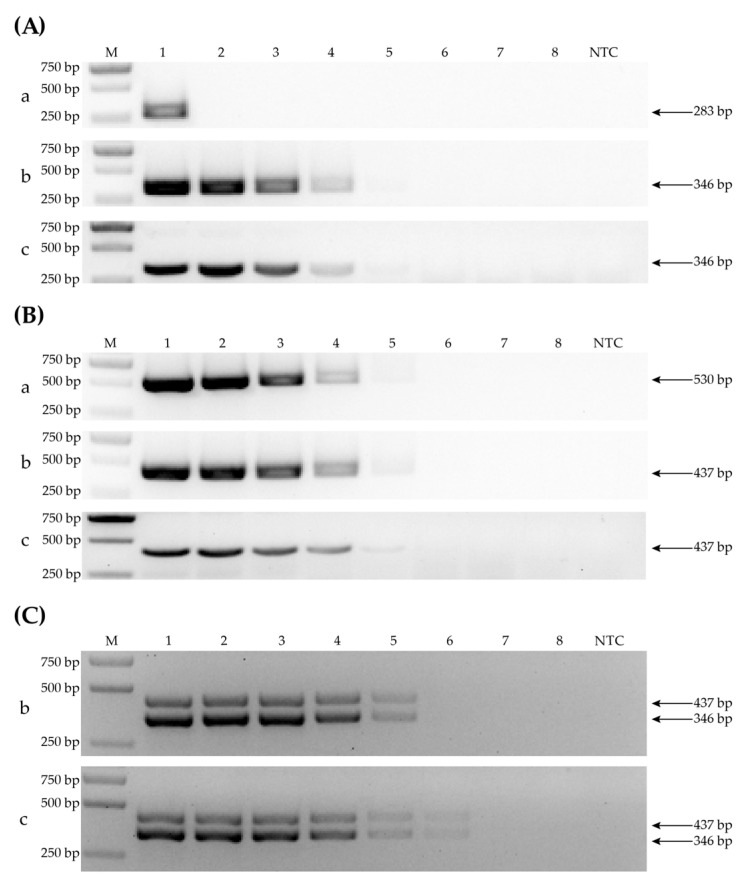
Viral amplification products using PCR-CP (a), PCR-RPA (b) and a handheld RT-RPA (c) to assess the sensitivity of the RT-RPA method. (**A**) Comparison of WYMV detection sensitivity levels. Lanes 1 to 8 are a high-to-low RNA concentration of WYMV in 10-fold serial dilution ranging from 100 to 10^–5^ ng/μL. (**B**) Comparison of CWMV detection sensitivity levels. The distribution of the lanes in the template is the same as that in (**A**). (**C**) Comparison of multiplex PCR-RPA and handheld RT-RPA. The distribution of the lanes in the template is the same as that in (**A**).

In order to assess the sensitivity of RPA to single or simultaneous detection of WYMV and CWMV by PCR-CP, PCR-RPA and RT-RPA, the initial RNA concentration of the three positive samples (HN-3 infected with WYMV, tobacco infected with CWMV, SD-15 co-infected CWMV and WYMV) was set to 100 ng/μL, and then serially 10-fold diluted to 10, 10^0^, 10^−1^, 10^−2^, 10^−3^, 10^−4^ and 10^−5^. For the detection of WYMV, RT-RPA and PCR-RPA showed the same level of sensitivity, but PCR-CP only amplified the target band in undiluted template (Figure 5A), which indicate that the primers of RT-RPA are more sensitive than primers of PCR-CP for WYMV. For the detection of CWMV, all three methods showed suitable levels of sensitivity (Figure 5B). For simultaneous detection of WYMV and CWMV, the RT-RPA method still detected two viral amplicons when the template was diluted to 10^−3^, while PCR-RPA did not (Figure 5C). This indicates that RT-RPA is more sensitive than PCR-RPA in the simultaneous detection of two viruses. 

### 3.6. Specificity Determination of RT-RPA Method

**Figure 6:**
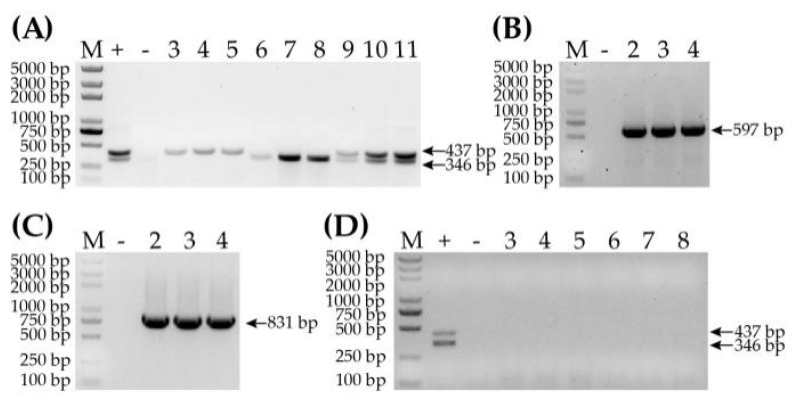
Specificity test of the RT-RPA method. (**A**) Amplified products of samples co-infected and singly infected with WYMV and CWMV. Lane M, D2000 Plus Ladder; +, positive control, a mixture of pCB-35S-R2 of CWMV and pCB-35S-R1 of WYMV plasmids; −, negative control, a healthy sample (SD-13); lanes 3 to 5 are tobacco samples infected with CWMV; lanes 6 to 8 are samples HN-3, HN-5 and SD-1, infected with WYMV; lanes 9 to 11 are samples SD-16, AH-4 and AH-6, co-infected with CWMV and WYMV. (**B**) PCR products of BSMV plasmid detection. Lane M, D2000 Plus Ladder; −, negative control, a healthy sample cultivated in laboratory; lanes 2 to 4 are plasmids of BSMV. (**C**) PCR products of WSMV plasmid detection. Lanes M and − are the same as in (**B**); lanes 2 to 4 are plasmids of WSMV. (**D**) Two-virus detection of WYMV and CWMV in BSMV and WSMV plasmids. Lanes M and + are same as in (**A**); −, negative control, a healthy sample cultivated in laboratory; lanes 3 to 5 are products of BSMV plasmid detection; lanes 6 to 8 are products of WSMV plasmid detection.

Given that WYMV and CWMV co-infect wheat naturally, and other common wheat viruses could infect wheat simultaneously, determining the specificity of the RT-RPA method for WYMV and CWMV is important. For testing the specificity of RT-RPA for the detection of WYMV and CWMV, selected RPA primers were tested against three samples with WYMV infection, three samples with CWMV infection, three samples with WYMV and CWMV co-infection and three recombinant plasmids of non-target BSMV or WSMV. The results showed that there were two obvious specific bands of the target fragments for co-infected samples and one band for samples with a single virus infection (Figure 6A), suggesting that RT-RPA had exceptional amplification efficiency when WYMV and CWMV were detected simultaneously in the same tube. In addition, the plasmids with cDNA sequences of BSMV and WSMV were detected using the specific primers of the two viruses, and the unique bands of BSMV or WSMV were clearly visible in gel (Figure 6B,C). Then, the plasmids with cDNA sequences of BSMV and WSMV were used as template to detect WYMV or CWMV, and the result showed that there were no bands corresponding to the target amplicons of WYMV or CWMV (Figure 6D). All these indicated that a two-virus-detecting RPA could be used to specifically detect samples with a single infection or co-infection of WYMV and CWMV.

### 3.7. Applicability of the RT-RPA Method

To verify that our RT-RPA method has credible applicability, 110 field samples were tested using RPA and the results were compared with a standard PCR (Supplementary Figures S1–S3, Table 2). We analyzed 57 samples infected with WYMV, 25 co-infected samples and 28 healthy samples using a standard PCR, and we analyzed 63 samples infected with WYMV, 22 co-infected and 25 healthy samples using RPA. The kappa value showed that the agreement rate between standard PCR and RPA was 0.926 (0.843~1.000, 95% confidence index (CI)) for WYMV and 0.919 (0.828~1.000, 95% CI) for CWMV. The sensitivity and specificity levels achieved were 100.0% (95.6%~100.0%, 95% CI) and 89.3% (71.8~97.7%, 95% CI), 88.0% (68.8~97.5%, 95% CI) and 100% (95.8~100.0%, 95% CI) for WYMV and CWMV detection, respectively (Table 3). This result indicated that the two-virus-detecting RPA method had sufficient agreement with the standard PCR method.

## 4. Discussion

As a rapid, sensitive, and specific isothermal amplification method, RPA has a short reaction time and is cheaper and more convenient than LAMP [28,37]. In this study, we also showed that RPA enzymes are able to perform primer annealing and elongation effectively at a 37 °C handheld temperature and in a 40 °C water bath, making this technology suitable for use in the absence of instruments [14]. However, improvements are needed to reduce operational cross-contamination and to improve the visualization of the RPA amplicons. In order to reduce the probability of cross-contamination and to save time cleaning products, there are many workable solutions, such as using a combination of RT-RPA and CRISPR/Cas12a. This would not only enable reverse transcription and the RPA reaction to be performed in one tube, thereby avoiding the likelihood of cross-contamination, but also would detect RPA products by capturing signals using a fluorescence viewer [37]. Another solution was the use of quantitative RT-RPA applied probes, which would visualize the amplification of the target gene by emitting a signal [30].

During the optimization of the WYMV and CWMV RT-RPA detection assay, we found that PCR-CP only detected the WYMV amplicon of the undiluted template (Figure 5A). This indicated that the primers of PCR-CP were relatively insensitive compared with the primers of RT-RPA for low WYMV load detection. In this study, PCR-CP only detects single virus whereas RPA was able to detect the corresponding virus in plants with either a single infection or in co-infected plants. We tested 110 field samples from five provinces in China using PCR-CP, PCR-RPA and a two-virus-detecting RPA. The results showed that the 95% confidence interval of the kappa values between standard PCR and PCR-RPA in WYMV and CWMV detection were both greater than 0.8, indicating that the results obtained with RPA mirrored those achieved using standard PCR [38]. This was sufficient to support the theoretical usage of RPA under field conditions. However, there was one false-positive result in the RPA-WYMV analysis, which may be the result of cross-contamination between WYMV and CWMV during the experimental operation. Nevertheless, this probability would be reduced in the field. As to whether any false-negative results were obtained in the RPA-CWMV analysis, it is possible that weak CWMV bands were too close to strong WYMV bands to be observed when the gels were visualized under UV light, which could explain the relatively weak sensitivity of the RPA-CWMV analysis. 

As a whole, our optimized RPA method is specific and sensitive and could be used for the simultaneous detection of WYMV and CWMV. Conventional wheat virus detection methods have strict requirements for sample storage and detection instruments. If we combined the RNA extraction methods at ambient temperature (such as crude leaf extracts) [36,39] and lateral flow dipstick assay [5] or the CRISPR/Cas12a system, and used RPA kits with reverse transcriptase [40], it would be possible to detect timely and early WYMV and CWMV through one-step RT-RPA in a field environment. Therefore, this study represents a significant step forward in wheat disease detection using the RPA method, enabling the early diagnosis, detection and characterization of wheat viruses.

## 5. Conclusions

Virus detection plays a significant role in disease prevention and control. The RT-RPA method simultaneously detecting wheat yellow mosaic virus and Chinese wheat mosaic virus has potential to be utilized in the field. After optimizing the reaction conditions, the handheld RT-RPA method show suitable sensitivity and specificity in the simultaneous detection of WYMV and CWMV. Our study is valuable to devote a theoretical basis and practical meaning to the handheld detection of WYMV and CWMV in the field.

## Figures and Tables

**Table 1:** Primer pairs designed for WYMV and CWMV RPA detection.

Primer Name	Sequence 5’-3’	Set	Targeted Region	Expected Product Length (bp)
WYMV-RPA-1-F	TTGACTTCTTTGTCCCCCGCCCGTGGATG	WYMV-S1	WYMV coat protein	166
WYMV-RPA-1-R	TGTCCATCAGTATCCAGAACCCTGCGGTTG			
WYMV-RPA-2-F	TGAATGGTGGCTTGAGACGGATCCTGCGAG	WYMV-S2	WYMV coat protein	250
WYMV-RPA-2-R	AGGTTGGTTGTGTCGGAGGTGAGCATGGTAT			
WYMV-RPA-3-F	CTTCAGACAAACACGCAGAAAACCAGACCA	WYMV-S3	WYMV coat protein	346
WYMV-RPA-3-R	TTGGTTGTGTCGGAGGTGAGCATGGTATTGT			
WYMV-RPA-4-F	ACCAGTCTTGGCATCACCACGGATGAGGCA	WYMV-S4	WYMV coat protein	445
WYMV-RPA-4-R	GGTTGGTTGTCTTGCGGAGGTTGGTTGTGT			
CWMV-RPA-1-F	CTTTTGCGACCTTGGCGGAGAGGGGGTCAGT	CWMV-S1	CWMV coat protein	158
CWMV-RPA-1-R	CAGTCTGCCCTTGTTCTTCTGTTTCTATCT			
CWMV-RPA-2-F	TAATTTCTTCTCACGGAGTAAACGCTTCGGT	CWMV-S2	CWMV coat protein	236
CWMV-RPA-2-R	TAGATATAGCCAACGATTGATCAGTCTGCCCTT			
CWMV-RPA-3-F	TGGCAGAGTACGAGGACGAGTGTGTTGTCT	CWMV-S3	CWMV coat protein	343
CWMV-RPA-3-R	AACTAACATAAGTCATCAGTTCGCCCAAAGCAT			
CWMV-RPA-4-F	AAGTTGAGACATGGCAGAGTACGAGGACGA	CWMV-S4	CWMV coat protein	437
CWMV-RPA-4-R	TCAACTCGAACCTTCCCACTTAAGATTATACT			

**Table 2:** Detection of WYMV and CWMV in field samples using PCR and RPA.

Sample	PCR		RPA		Sample	PCR		RPA	
	WYMV	CWMV	WYMV	CWMV		WYMV	CWMV	WYMV	CWMV
Henan-1	+	+	+	-	Anhui-1	+	-	+	-
Henan-2	+	-	+	-	Anhui-2	+	-	+	-
Henan-3	+	-	+	-	Anhui-3	+	-	+	-
Henan-4	+	-	+	-	Anhui-4	+	-	+	-
Henan-5	+	-	+	-	Anhui-5	+	-	+	-
Henan-6	+	-	+	-	Anhui-6	+	+	+	+
Henan-7	-	-	-	-	Anhui-7	+	+	+	+
Henan-8	-	-	-	-	Anhui-8	+	+	+	+
Henan-9	-	-	-	-	Anhui-9	-	-	-	-
Henan-10	+	-	+	-	Anhui-10	-	-	-	-
Henan-11	+	-	+	-	Anhui-11	-	-	-	-
Henan-12	+	-	+	-	Anhui-12	-	-	-	-
Henan-13	+	-	+	-	Anhui-13	-	-	-	-
Henan-14	+	-	+	-	Anhui-14	-	-	-	-
Henan-15	+	-	+	-	Anhui-15	-	-	-	-
Henan-16	+	-	+	-	Anhui-16	-	-	-	-
Henan-17	-	-	-	-	Anhui-17	-	-	-	-
Henan-18	-	-	-	-	Shandong-1	+	+	+	+
Henan-19	-	-	-	-	Shandong-2	+	-	+	-
Henan-20	-	-	-	-	Shandong-3	+	+	+	-
Henan-21	-	-	-	-	Shandong-4	-	-	-	-
Henan-22	-	-	-	-	Shandong-5	-	-	-	-
Henan-23	-	-	-	-	Shandong-6	+	+	+	+
Henan-24	-	-	-	-	Shandong-7	+	+	+	+
Henan-25	-	-	-	-	Shandong-8	+	+	+	+
Henan-26	+	-	+	-	Shandong-9	+	+	+	+
Henan-27	+	-	+	-	Shandong-10	+	+	+	-
Henan-28	+	-	+	-	Shandong-11	+	+	+	+
Henan-29	+	-	+	-	Shandong-12	+	+	+	+
Henan-30	+	-	+	-	Shandong-13	+	+	+	+
Henan-31	+	-	+	-	Shandong-14	+	+	+	+
Henan-32	+	-	+	-	Shandong-15	+	-	+	-
Henan-33	+	-	+	-	Shandong-16	+	-	+	-
Henan-34	+	-	+	-	Shandong-17	+	-	+	-
Henan-35	+	-	+	-	Shandong-18	+	+	+	+
Henan-36	+	-	+	-	Shandong-19	+	+	+	+
Henan-37	+	-	+	-	Shandong-20	+	+	+	+
Henan-38	+	-	+	-	Shandong-21	+	+	+	+
Henan-39	+	-	+	-	Shandong-22	+	+	+	+
Henan-40	+	-	+	-	Shandong-23	+	+	+	+
Henan-41	+	-	+	-	Shandong-24	+	+	+	+
Henan-42	+	-	+	-	Shandong-25	+	+	+	+
Shanxi-1	+	-	+	-	Shandong-26	+	+	+	+
Shanxi-2	-	-	-	-	Shandong-27	+	+	+	+
Shanxi-3	-	-	+	-	Shandong-28	+	-	+	-
Shanxi-4	-	-	-	-	Shandong-29	+	-	+	-
Shanxi-5	+	-	+	-	Shandong-30	+	-	+	-
Shanxi-6	+	-	+	-	Shandong-31	+	-	+	-
Shanxi-7	-	-	+	-	Jiangsu-1	-	-	+	-
Shanxi-8	+	-	+	-	Jiangsu-2	+	-	+	-
Shanxi-9	+	-	+	-	Jiangsu-3	+	-	+	-
Shanxi-10	+	-	+	-	Jiangsu-4	+	-	+	-
Shanxi-11	+	-	+	-	Jiangsu-5	+	-	+	-
Shanxi-12	+	-	+	-	Jiangsu-6	+	-	+	-
Shanxi-13	+	-	+	-	Jiangsu-7	+	-	+	-

**Table 3:** Detection performance comparison between standard PCR and RT-RPA assays.

		**PCR-CP^a^**			**Performance Characteristics (%)**	
		**Positive**	**Negative**	**Total**	**Sensitivity**	**Specificity**
**RPA-** **WYMV^c^**	Positive	82	3	85	100.0% (95.6~100.0%, 95% CI)	89.3% (71.8~97.7%, 95% CI)
	Negative	0	25	25		
		82	28	110		
Agreement Kappa value: 0.926 (0.843~1.000, 95% CI)						
		**PCR-CP^b^**			**Performance Characteristics (%)**	
		**Positive**	**Negative**	**Total**	**Sensitivity**	**Specificity**
**RPA-** **CWMV^d^**	Positive	22	0	22	88.0%(68.8~97.5%, 95% CI)	100% (95.8~100.0%, 95% CI)
	Negative	3	81	84		
		25	81	106		
Agreement Kappa value: 0.919 (0.828~1.000, 95% CI)						

PCR-CP^a^ represents detection of WYMV by PCR with common CP primers; PCR-CP^b^ represents detection of CWMV with common CP primers; RPA-WYMV^c^ represents detection of WYMV by handheld RT-RPA; RPA-CWMV^d^ represents detection of CWMV by handheld RT-RPA.

## Data Availability

Not applicable.

## Supplementary Materials

The following supporting information can be downloaded at: https://www.mdpi.com/article/10.3390/life12121952/s1, Figure S1: The detection WYMV using PCR-CP. Figure S2: The detection of CWMV using PCR-CP. Figure S3: The simultaneous detection of WYMV and CWMV using handheld RT-RPA. Table S1: ID of CP-encoding gene sequence of CWMV and WYMV. Table S2: Primer pairs used for WYMV, CWMV, BSMV and WSMV PCR detection.
