# Supplementary material for: Rapid, Sensitive and Simultaneous Detection of Two Wheat RNA Viruses Using Reverse Transcription Recombinase Polymerase Amplification (RT-RPA)
Source: Life (Basel). 2022 Nov 22;12(12):1952. doi: 10.3390/life12121952 (PMC9788578; doi:10.3390/life12121952)
Supplement: Supplementary file 1 [file life-12-01952-s001.zip › Supplemental tables-revised.pdf]

Table S1. ID of CP encoding gene sequence of CWMV and WYMV

Virus	GenBank accession number
WYMV	D86634.1, AB627819.1, AB627818.1, AB948222.1, AB627815.1, AB627814.1, AB627817.1, AB627813.1, AB627812.1, FR827993.1, LC404158.1, LC484284.1, FR828159.1, FR828154.1, FR828153.1, FR828145.1, FR828069.1, FR827999.1, AJ243982.1, AB627816.1, FR828178.1, FR828064.1, FR828027.1, FR828017.1, FR828013.1, FR828010.1, FR828006.1, FR828005.1, FR828004.1, FR828003.1, FR827998.1, AJ243985.1, FR828079.1, FR828028.1, FR828026.1, FR828023.1, FR828014.1, FR827994.1, FR827981.1, AJ131981.1, MG678348.1, MG678326.1, KX258948.1, AB910330.1, AB910329.1, FR828071.1, FR828061.1, FR828008.1, FR827986.1, FR827980.1, AJ243983.1, AJ240051.1, MG678322.1, MG678338.1, MG678334.1, FR828078.1, FR828075.1, FR828067.1, FR828056.1, FR828046.1, FR828036.1, FR827976.1, FJ361764.1, AJ243988.1, AJ239038.1, MG678346.1, MG678345.1, MG678344.1, LC404159.1, AB910332.1, AB627810.1, AB627807.1, AB627806.1, FR828120.1, FR828080.1, FR828049.1, FR828038.1, FR827984.1, FR827978.1, FR827946.1, FJ361766.1, AJ243953.1, AJ243987.1, AJ243986.1, AJ240048.1, MG678349.1, MG678337.1, MG678331.1, MG678323.1, AB910331.1, FR828210.1, FR828209.1, FR828180.1, FR828161.1, FR828127.1, FR828119.1, FR828076.1, FR828073.1, FR828070.1, FR828044.1
CWMV	AB935554.1, AJ012006.2, AB299272.1, AJ271839.1, EF121374.1

Table S2. Primer pairs used for WYMV, CWMV, BSMV and WSMV PCR detection

Primer Name	Sequence 5'-3'	Targeted region	Expected product length (bp)
WYMV-CP-F	ATGATGCCGACGCTGCGCGTC	WYMV coat protein	283 bp
WYMV-CP-R	CTGGAGTTGTTGTGTTCGATT		
CWMV-CP-F	ATGGCCGTGAAATCTGGTTAT	CWMV coat protein	530 bp
CWMV-CP-R	ACACTCGAACCTTCCCACTTAAG		
BSMV-CP-F	ATGCCGAACGTTTCTTTGAC	BSMV coat protein	597 bp
BSMV-CP-R	TCACGCTTCCTCGGCATCTG		
WSMV-CP-F	CTGGTTGACACGCTATCTCC	WSMV polyprotein	831 bp
WSMV-CP-R	TGGTTTCTCATCATGGCACT		
