# Supplementary material for: Rapid, Sensitive and Simultaneous Detection of Two Wheat RNA Viruses Using Reverse Transcription Recombinase Polymerase Amplification (RT-RPA)
Source: Life (Basel). 2022 Nov 22;12(12):1952. doi: 10.3390/life12121952 (PMC9788578; doi:10.3390/life12121952)
Supplement: Supplementary file 1 [file life-12-01952-s001.zip › Supplementary Figures-revised.pdf]

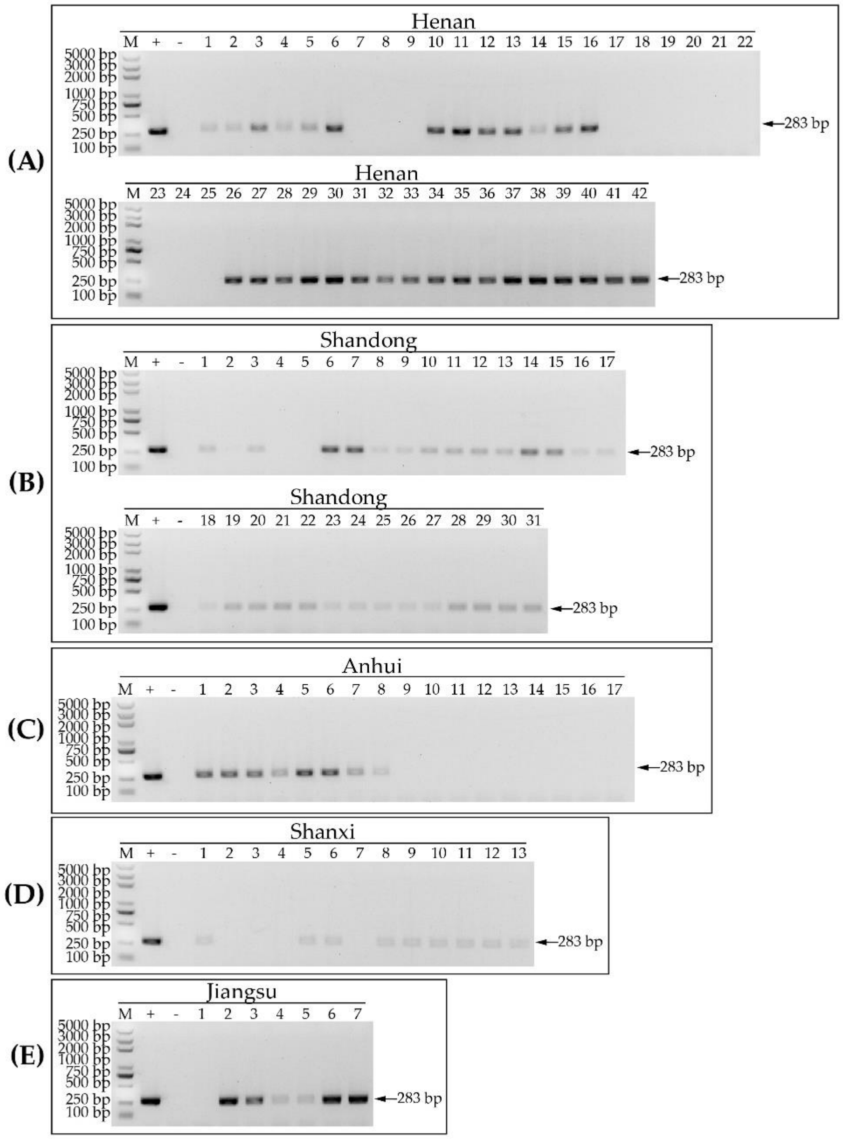

Supplementary Figure S1. The detection of WYMV using PCR-CP. **(A)** The detection of WYMV in 42 samples from Henan province (lane M, D2000 Plus Ladder; +, positive control, pCB-35S-R1 of WYMV plasmids; –, negative control, a healthy sample (SD-13); lanes 1-42 are samples Henan-1 to Henan-42, respectively). **(B)** The detection of WYMV in 31 samples from Shandong province (lane M, +, and – are the same as those in Figure S1A; lanes 1-31 are samples Shandong-1 to Shandong-31, respectively). **(C)** The detection of WYMV in 17 samples from Anhui province (lane M, +, and – are the same as those in Figure S1A; lanes 1-17 are samples Anhui-1 to Anhui-17, respectively). **(D)** The detection of WYMV in 13 samples from Shanxi province (lane M, +, and – are the same as those in Figure S1A; lanes 1-13 are samples Shanxi-1 to Shanxi-13, respectively). **(E)** The detection of WYMV in 7 samples from Jiangsu province (lane M, +, and – are the same as those in Figure S1A; lanes 1-7 are samples Jiangsu-1 to Jiangsu-7, respectively).

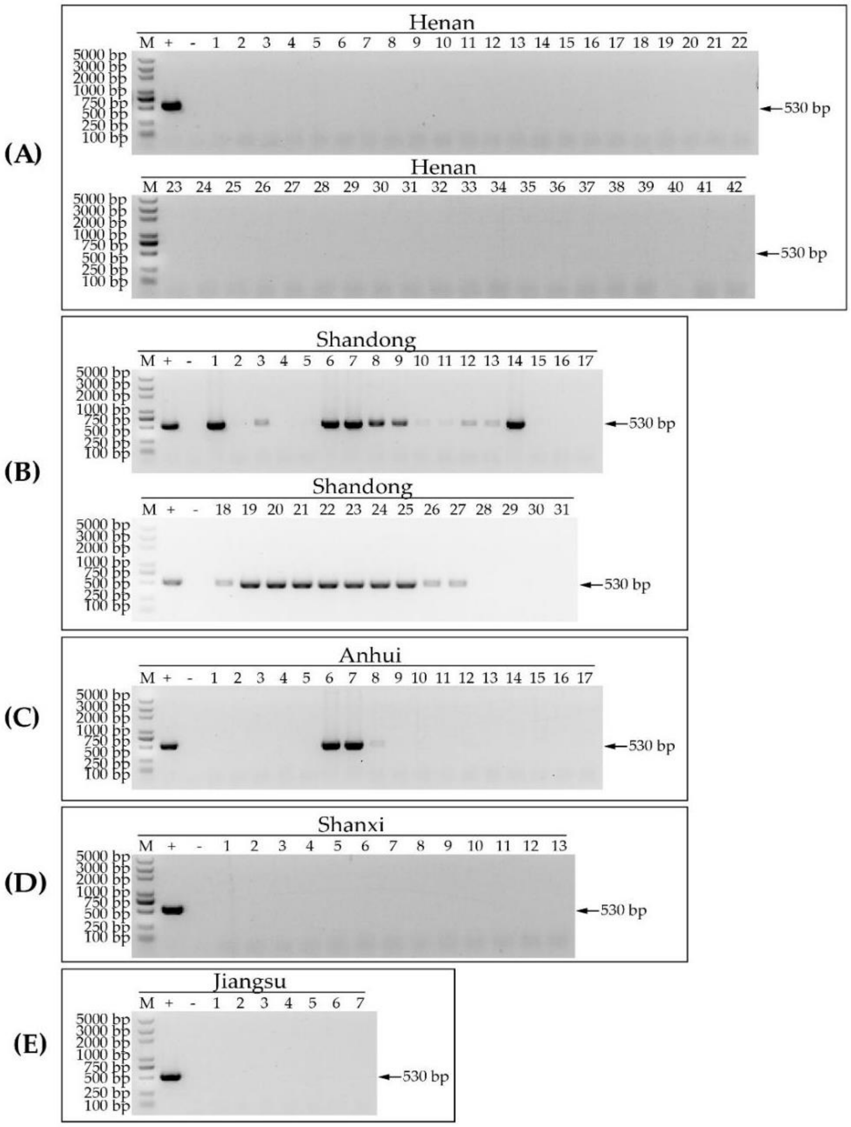

Supplementary Figure S2. The detection of CWMV using using PCR-CP. **(A)** The detection of CWMV in 42 samples from Henan province (lane M, D2000 Plus Ladder; +, positive control, pCB-35S-R2 of CWMV plasmids; –, negative control, a healthy sample (SD-13); lanes 1-42 are samples Henan-1 to Henan-42, respectively). **(B)** The detection of CWMV in 31 samples from Shandong province (lane M, +, and – are the same as those in Figure S2A; lanes 1-31 are samples Shandong-1 to Shandong-31, respectively). **(C)** The detection of CWMV in 17 samples from Anhui province (lane M, +, and – are the same as those in Figure S2A; lanes 1-17 are samples Anhui-1 to Anhui-17, respectively). **(D)** The detection of CWMV in 13 samples from Shanxi province (lane M, +, and – are the same as those in Figure S2A; lanes 1-13 are samples Shanxi-1 to Shanxi-13, respectively). **(E)** The detection of CWMV in 7 samples from Jiangsu province (lane M, +, and – are the same as those in Figure S2A; lanes 1-7 are samples Jiangsu-1 to Jiangsu-7, respectively).

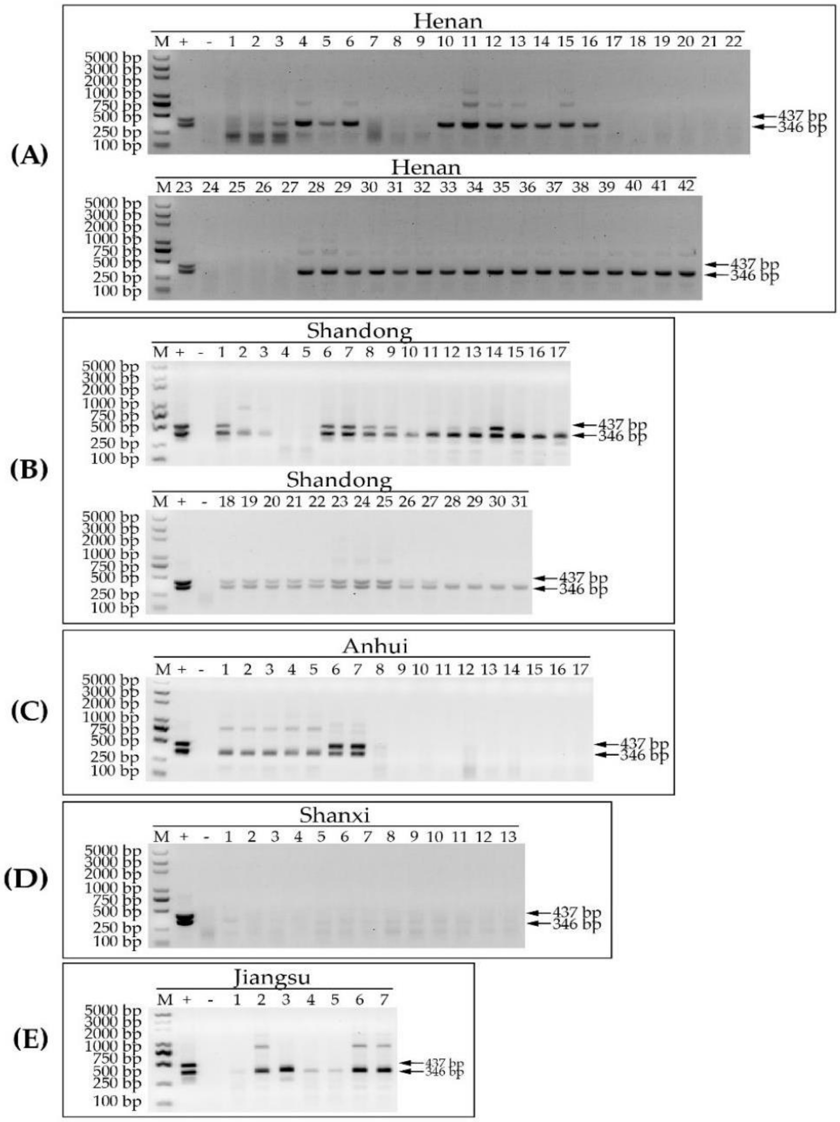

Supplementary Figure S3. The simultaneous detection of WYMV and CWMV using handheld RT-RPA. Simultaneous detection of WYMV and CWMV (lane M, D2000 Plus Ladder; +, positive control, a mixture of pCB-35S-R2 of CWMV and pCB-35S-R1 of WYMV plasmids; –, negative control; lanes 1-42 are samples Henan-1 to Henan-42, respectively). (B) Simultaneous detection of WYMV and CWMV in 31 samples from Shandong province (lane M, +, and – are the same as those in Figure S3A; lanes 1-31 are samples Shandong-1 to Shandong-31, respectively). (C) Simultaneous detection of WYMV and CWMV detection in 17 samples from Anhui province (lane M, +, and – are the same as those in Figure S3A; lanes 1-17 are samples Anhui-1 to Anhui-17, respectively). (D) Simultaneous detection of WYMV and CWMV in 13 samples from Shanxi province (lane M, +, and – are the same as those in Figure S3A; lanes 1-13 are samples Shanxi-1 to Shanxi-13, respectively). (E) Simultaneous detection of WYMV and CWMV in 7 samples from Jiangsu province (lane M, +, and – are the same as those in Figure S3A; lanes 1-7 are samples Jiangsu-1 to Jiangsu-7, respectively).).
